# A comparison of herbarium and citizen science phenology datasets for detecting response of flowering time to climate change in Denmark

**DOI:** 10.1007/s00484-022-02238-w

**Published:** 2022-03-02

**Authors:** Natalie Iwanycki Ahlstrand, Richard B. Primack, Anders P. Tøttrup

**Affiliations:** 1 Natural History Museum of Denmark, University of Copenhagen, Copenhagen, Denmark; 2 Biology Department, Boston University, Boston, MA USA

**Keywords:** Phenology, Herbarium specimens, Citizen science, Flowering, Climate change, Spatiotemporal variation

## Abstract

Phenology has emerged as a key metric to measure how species respond to changes in climate. Innovative means have been developed to extend the temporal and spatial range of phenological data by obtaining data from herbarium specimens, citizen science programs, and biodiversity data repositories. These different data types have seldom been compared for their effectiveness in detecting environmental impacts on phenology. To address this, we compare three separate phenology datasets from Denmark: (i) herbarium specimen data spanning 145 years, (ii) data collected from a citizen science phenology program over a single year observing first flowering, and (iii) data derived from incidental biodiversity observations in iNaturalist over a single year. Each dataset includes flowering day of year observed for three common spring-flowering plant species: *Allium ursinum* (ramsons), *Aesculus hippocastanum* (horse chestnut), and *Sambucus nigra* (black elderberry). The incidental iNaturalist dataset provided the most extensive geographic coverage across Denmark and the largest sample size and recorded peak flowering in a way comparable to herbarium specimens. The directed citizen science dataset recorded much earlier flowering dates because the program objective was to report the first flowering, and so was less compared to the other two datasets. Herbarium data demonstrated the strongest effect of spring temperature on flowering in Denmark, possibly because it was the only dataset measuring temporal variation in phenology, while the other datasets measured spatial variation. Herbarium data predicted the mean flowering day of year recorded in our iNaturalist dataset for all three species. Combining herbarium data with iNaturalist data provides an even more effective method for detecting climatic effects on phenology. Phenology observations from directed and incidental citizen science initiatives will increase in value for climate change research in the coming years with the addition of data capturing the inter-annual variation in phenology.

**Supplementary Information:**

The online version contains supplementary material available at 10.1007/s00484-022-02238-w.

## Introduction

Phenology has emerged as a key indicator of how species respond to climate change (Cleland et al. 2007; Tang et al. 2016). However, scientific data tracking phenology over time are often limited in spatial and temporal scope. Most phenology studies involve long-term monitoring at only a single location, though some phenological networks, such as the International Phenological Gardens of Europe programme, monitor numerous stations (Sparks et al. 2000; Estrella et al. 2009; Fitter and Fitter 2002; Menzel 2003; Taylor et al., 2019; Fox and Weisberg, 2019; IPG, 2021). To overcome the sparsity of long-term and spatially widespread phenology datasets, scientists have been developing innovative new methods (Cleland et al. 2007). For example, herbarium specimens are increasingly used to recreate long-term phenology observations of flowering, leaf out, and fruiting over the past 100–150 years and over broad geographical regions (Primack et al. 2004; Willis et al. 2017). Meanwhile, directed citizen science initiatives and community biodiversity data repositories such as iNaturalist are providing new sources of phenology data covering broad geographic regions (iNaturalist, 2021). These new phenology data sources are growing fast; herbarium specimen digitization is happening at unprecedented rates, and the expansion of new citizen science phenology initiatives is occurring rapidly (Belitz et al. 2020). Both herbarium-derived and citizen science phenology data have the potential to cover vast geographic regions and therefore have enormous potential for informing species-specific responses to climate change.

These various phenology data sources come along with advantages and limitations for climate change research (Davis et al., 2015; Willis et al. 2017; Taylor et al. 2019; Funk 2018; Belitz et al. 2020). Most notably, herbarium specimens and citizen science networks include biases in sampling locations, taxonomic groups, and gaps in sampling years over time (Delisle et al. 2003; Daru et al. 2018; Panchen et al. 2019). Also, many historical specimens lack precise locality information, presenting challenges for accurate georeferencing and analysis (Maldonado et al. 2015; Bloom et al. 2017). The digitization process itself may introduce further error or biases for phenology or other data derived from digitized herbarium specimens (Groom et al. 2019). Due to these limitations, supplementing phenology data derived from herbarium specimens with other types of phenology data is proving to be potentially valuable approach (Davis et al. 2015; Spellman and Mulder 2016; Jones and Daehler 2018).

Citizen science networks, in particular, have the potential to provide enormous numbers of phenological observations over huge geographic areas, filling in gaps not covered by herbarium specimens (Willis et al. 2017). With limited training, citizen scientists of a wide range of backgrounds can provide reliable phenology observations (Fuccillo Battle et al. 2014; Bison et al. 2019), including observations on private lands which would otherwise be inaccessible to researchers (Putman et al. 2021). In some cases, phenology data collected by citizen scientists have been known to suffer from poor data quality particularly in terms of species identification, as well as the drop out of participants over time which poses challenges for the successful establishment of long-term citizen science monitoring networks (Beaubien and Hamann 2011; Fuccillo Battle et al. 2014; MacKenzie et al. 2017). The main limitation of using citizen science datasets in climate change research is the number of years of observations is still very limited.

Community-driven biodiversity data repositories such as iNaturalist, while not explicitly targeted at phenology monitoring, also provide a huge potential source of phenology data, as people have uploaded millions of photographs of plants in flower for identification purposes (Heberling and Isaac 2018; Putman et al. 2021; iNaturalist, 2021). These photographs also provide digital vouchers that can be used to confirm species identity, which is a problem with unverified citizen science observations.

These various new and traditional phenology approaches are increasingly regarded as complementary because they cover different spatial and temporal scales (Spellman and Mulder 2016; Willis et al. 2017). However, they have seldom been quantitatively compared for their ability to detect the impacts of climate change on phenology (Taylor et al., 2019). We address this gap with an explicit quantitative comparison of three phenology datasets for their relative sensitivities in detecting the response of flowering to climate over time and space in Denmark: (i) phenology data derived from herbarium specimens spanning 145 years (“herbarium dataset,” *n* = 110), (ii) data from a directed phenology citizen science project collected from a single year (“citizen science dataset,” *n* = 104), and (iii) phenology data derived from incidental biodiversity observations using iNaturalist over a single year (“iNaturalist dataset,” *n* = 403). Each dataset includes flowering day of year observed for three spring-flowering plant species common throughout Denmark: *Allium ursinum* L. (ramsons), *Aesculus hippocastanum* L. (horse chestnut), and *Sambucus nigra* L. (black elderberry). The goal of the study is to determine if these three different datasets are equally effective at detecting the spatial and temporal effects of climate change and test whether they can be easily combined to improve statistical predictions. Each dataset has certain advantages: the herbarium data set covers the longest time span, the directed citizen science data set was established specifically for phenology research, and the iNaturalist data set has the largest sample size. We explore spatial and temporal relationships between temperature and flowering time and test whether phenological datasets with wide geographical coverage can be a surrogate to phenological data spanning long time periods.

## Materials and methods

### Study region

Denmark is a small country (42,933 km^2^) with a varied landscape and coastal climate (Lawesson and Skov 2002). The country is relatively flat, with a maximum elevation of 170 m above sea level and minimal microclimate effects associated with local topography. Denmark has a long history of climate data archived by the Danish Meteorological Institute, relatively large collections of herbarium specimens, and numerous citizens participating in recent phenology initiatives.

### Study species

Three common spring-flowering vascular plant species, *Allium ursinum*, *Aesculus hippocastanum*, and *Sambucus nigra*, were selected as model species based on the relative abundance of flowering phenology data from herbarium records, new citizen science programs, and iNaturalist observations (Table 1). Previous phenological research from Europe has demonstrated that the phenologies of these species are responsive to climate change (e.g. Sparks et al. 2000; Defila and Clot 2001; Menzel 2003; Szabó et al. 2016; Heinrichs et al. 2018; Fox and Weisberg, 2019; Timberlake et al. 2019).

**Table 1:** Comparison of mean flowering dates (± standard deviation, SD) between herbarium, citizen science, and iNaturalist phenology datasets. ANOVA results (*F*-value, *p*-value) are based on comparing the mean flowering day of year (DOY) for each dataset

*Species*	Dataset	*n*	Earliest–latest DOY	Mean flowering DOY ± SD	ANOVA *F*-value	ANOVA *P*-value
*Allium ursinum*	Herbarium	57	105–183	150 ± 13.9	151.6	< 0.001
	Citizen science	19	79–116	103 ± 12.2		
	iNaturalist	183	103–153	133 ± 9.0		
*Aesculus hippocastanum*	Herbarium	14	129–198	153 ± 10.2	60.3	< 0.001
	Citizen science	52	117–150	133 ± 7.7		
	iNaturalist	33	124–154	141 ± 7.0		
*Sambucus nigra*	Herbarium	39	105–194	183 ± 16.5	78.7	< 0.001
	Citizen science	33	124–167	151 ± 9.2		
	iNaturalist	187	142–202	166 ± 9.6		

All three species have long histories of human use in Denmark. *Allium ursinum* is a herbaceous edible perennial monocot that grows in woodlands, with leaves that emerge in early spring, followed weeks later by inflorescences (Tutin 1957; Sobolewska et al. 2015). *Aesculus hippocastanum* is a large deciduous flowering tree cultivated throughout Denmark and with fruit used in handicrafts (Thomas et al. 2019). *Sambucus nigra* is a common shrub found in open fields and woodlands; its flowers and fruits are collected for food and medicinal use (Knudsen and Kaack  2015).

A recent atlas project for the flora of Denmark confirms how prevalent our focal plants are based on presence data from 5 × 5 km grid cells surveyed across the country (Hartvig 2015; Bruun Asmussen Lange and Hermann 2016). *Aesculus hippocastanum* and *Sambucus nigra* were reported from almost every atlas square surveyed in the Atlas Flora Danica project; *Allium* was also found to be widely distributed, but gaps in its distribution are evident in the very south and middle of the mainland (Jutland), matching the distribution of forest cover in the country (Hartvig 2015).

### Herbarium dataset

Herbarium specimens of the three focal species collected between 1839 and 1993 were examined at the Natural History Museum of Denmark (Herbarium C). For each specimen, we recorded the date of collection (day, month, and year; hereafter, day of the year), collection locality, and the topographical botanic unit (TBU) district number, a phytogeographical landscape classification system that divides Denmark into 57 floristic units (Lawesson and Skov 2002). The phenological stage for each specimen (bud, flowering, fruit, and vegetative/sterile) was assessed following a modified protocol of Yost et al. (2018). Specimens with only the flowering phenophase were retained and used in our analyses due to the sparsity of other phenological stages represented in the herbarium material. As almost all flowering specimens had most of the flowers open, we assumed flowering phenophase to mainly represent peak flowering (Jones and Daehler 2018). A recent study has shown that scoring flowering time as two or more flowering phases does not provide much additional insight in phenology studies using herbarium specimens (Ellwood et al. 2019). We omitted specimens lacking complete collection date records (day, month, and year), those collected before climate data was available, and specimens representing cultivars or from gardens. Specimens were manually georeferenced using geographic coordinates of the closest collection locality names and/or by using TBU district numbers in the case of ambiguous place names. The final filtered dataset included 14–57 flowering specimens per species collected between 1846 and 1991 (Table 1).

### Citizen science dataset

First flowering dates observed in 2020 for the three species were obtained from the phenology citizen science program *Danmark Udforsker* (Exploring Denmark) managed by the Natural History Museum of Denmark (www.danmarkudforsker.dk). There were 19–52 flowering observations per species and all observations were retained for analyses (Table 1). This program, launched in 2020, includes citizen science observations for over 50 phenological events, including our three focal plant species. Citizen scientists were recruited by the museum from across Denmark and invited to submit observations for a predetermined list of phenology events using a web-based app, which included precise geographical locations. For the program, a team of experts selected easily recognizable and broadly known species and phenological events to reduce the probability of misidentifications and ensure the broadest public appeal. As part of the instructions, photos and text presenting species biology and identification tips were provided; the program did not provide any direct training.

### iNaturalist dataset

A total of 982 iNaturalist research-grade records of the three species contributed from Denmark were downloaded from iNaturalist on January 13, 2021. Photographs linked to each iNaturalist record were reviewed to validate plant identity and date of observation. Phenophases were recorded for each observation, following the same protocol as described above for herbarium specimens. Records were filtered to include only flowering observations recorded in 2020, resulting in 33–187 flowering observations per species (Table 1). As with herbarium data, the assumption was made that each observation scored as flowering phenophase represents a species’ peak flowering since photographs clearly showed most flowers fully open.

### Climatic variables

Two climatic datasets were used in our analyses: historical climate data was matched with our historical herbarium dataset, and modern (higher resolution) climatic data was matched with our 2020 citizen science and iNaturalist datasets (Supplement information S1). Historical monthly weather data for the period 1768–2019 was obtained from the Danish Meteorological Institute (DMI) for five weather stations in Denmark (Cappelen et al., 2020). Average monthly temperatures for March, April, and May of the year of flowering were used in the analysis. Combining multiple months into a single climatic variable (i.e., average spring temperature) has previously been found to produce significant relationships between phenological events and climate and is thus commonly used based on the assumption that flowering DOY is affected by temperature in the months preceding flowering (Sparks et al., 2000; Menzel 2003; Primack et al. 2004; Jones and Daehler 2018). Using average temperatures also give results that are comparable to more complex degree day models (Balser, 2016) but has the added advantage of being more easily understood, which is an advantage in presenting the results to the public. Average spring temperature was therefore calculated by combining the monthly averages for March, April, and May. Each observation in our herbarium dataset was linked to its closest historical weather station and matched with the climate variables corresponding to the flowering day of year in the year of collection.

Hourly weather data from DMI for the months of March–May were downloaded from 45 available weather stations in 2020 using a python script run in Jupyter Notebook provided by DMI. Monthly averages were calculated in *R* v. 3.6.1 (R Core Team, 2019) to create the following weather parameters comparable to the historic climate data: average spring temperature (average of months of March, April, and May). Each observation in our citizen science and iNaturalist datasets were linked to the closest weather stations based on geographical coordinates, and corresponding climate data were assigned.

### Statistical analysis

To compare how each dataset varied with respect to the observed time of flowering, the mean flowering DOY was calculated in *R* for each species and each dataset. One-way ANOVAs and Tukey HSD post hoc tests were run to compare means for each species in each dataset.

Spatial distribution patterns between each dataset were analyzed to identify biases in the geographic distribution of flowering observations using the *spatsat* package in *R* (Baddely and Turner 2005). Geographic coordinates for each observation in each dataset were plotted, and the intensity of the observations and the two-dimensional kernel densities were calculated. Intensity measures the average density of points per unit area, and kernel density maps the probability density to visualize spatial biases (Baddely and Turner 2005). Observation point data from each of the three datasets were visually compared with data from Atlas Flora Danica (Hartvig 2015) to estimate spatial under-sampling based on known species distributions.

In order to test the sensitivity of each dataset in determining the response of flowering to temperature, we used both simple linear regressions and linear mixed models. Simple linear regressions were run individually for each species and each dataset, using “flowering day of year” as a response variable and climatic variables as predictor variables (average temperatures in the months of March, April, and May, and average spring temperature, combining the months into an average). Details are presented in Supplementary information (S3).

Separate linear mixed models were run for each of our three datasets (herbarium, citizen science, and iNaturalist) and a fourth dataset combining herbarium and iNaturalist data using the *lme4* package in *R* (Bates et al. 2015). We first assessed potential collinearity issues by conducting correlation analyses between climate (average spring temperature) and other predictor variables (longitude, latitude) using the Pearson cor.test in *R*. Due to the correlation between temperature and geographic variables in our data, separate models were run with either temperature and year (“temperature models”) or with geographical coordinates and year as fixed effects (“geographic models”). For all models, “flowering day of year” was the response variable, and “species” was treated as a random effect. For temperature models, “average spring temperature,” and in the case of the herbarium and combined datasets, “collection year” were fixed effects. For geographic models, latitude and longitude, and year were treated as fixed effects. Variance inflation factors (VIF) were calculated for each model using the *car* package in *R* to test for multicollinearity between variables where relevant (Fox and Weisberg, 2019), and a stringent approach of selecting a maximum VIF value of 2 was used to detect collinearity (Zurr et al. 2010). Best fit models for each dataset were determined by using maximum likelihood in lme4 (REML = FALSE) and then calculating the Akaike’s information criterion (AIC) value and choosing the model with the lowest AIC to determine the set of variables that best explain variation in flowering day of year (DOY). This was done separately for temperature models and for geographic models. Marginal and conditional *R*^2^ values for each model were calculated using the r.squaredGLMM functions in the *MuMIn* package (Bartoń, 2020). Marginal *R*^2^ values represent the variance explained by the fixed effects in the model, while conditional *R*^2^ is interpreted as the variance explained by both fixed and random effects (Nakagawa and Schielzeth 2013). *P*-values for fixed effects were calculated using the lmer.test in *R* (Kuznetsova et al. 2017), which we use to infer the probability of obtaining the coefficient under the null hypothesis of no effect. We assess the significance of our predictor variables at *p* < 0.05. Due to the thorough validation of observation data in each dataset and our goal to compare sensitivity between datasets, outliers were not omitted.

## Results

### Flowering day of year

Mean flowering day of year differed significantly between datasets for each of the three focal species based on ANOVA and Tukey HSD tests (Table 1). The herbarium dataset, which is assumed to represent peak flowering and covered the longest period of time, had the latest mean flowering DOY for each species (Table 1). The citizen science dataset recorded the earliest mean flowering DOY for each species, as these observations represent the first flowering DOY. These mean flowering dates for the citizen science data set are 3–7 weeks earlier than the herbarium datasets. The iNaturalist dataset was found to have a mean flowering DOY intermediate to the other two datasets, or 1.5 to 3 weeks earlier than the mean flowering DOY derived from herbarium observations, which likely is due to the advanced warm weather in the year 2020.

**Fig. 1:**
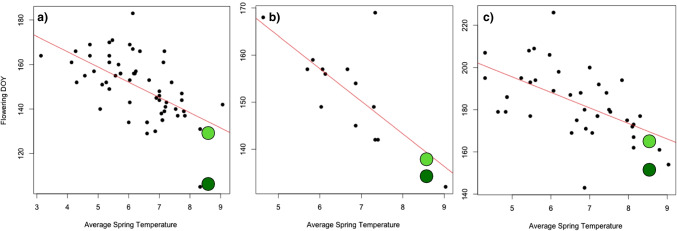
Herbarium data (for specimens collected between 1872 and 1993 predicts flowering day of year for iNaturalist data based on 2020 climatic data, but not for citizen science data (which recorded first flowering). x-axis: average spring temperatures based on means for the months of March, April, and May; *y*-axis: flowering day of year recorded from specimens. Regression lines shown in red. Light green points indicate mean flowering DOY for iNaturalist data and dark green points represent mean DOY for citizen science data. **a** *Allium ursinum* (*n* = 57), DOY = 193.19 +  − 6.9 T °C; *R*^2^ = 0.35, *F* = 29.4, *p* < 0.001. **b** *Aesculus hippocastanum* (*n* = 13), DOY = 198.73 +  − 6.9 T °C; *R*^2^ = 0.51, *F* = 12.44, *p* < 0.01; **c** *Sambucus nigra* (*n* = 35), DOY = 232.40 +  − 7.37 T °C; *R*^2^ = 0.32, *F* = 17.06, *p* < 0.001

For all three species, the relationship between temperature and mean flowering derived from our herbarium data predicted 2020 mean flowering DOY, which was very close to the actual day of year derived from iNaturalist data (Fig. 1). For example, in the case of *Allium*, a flowering DOY of 134 would be predicted based on the regression equation obtained from a simple linear regression of *Allium* flowering DOY and average spring temperature for the herbarium dataset (DOY = 193.189 +  − 6.853 T °C). This is very close to day 133 recorded in the iNaturalist dataset based on the recorded average spring temperature of 8.6 °C for March–May, 2020 (Table 1). Likewise, a flowering DOY of 139 would be predicted for *Aesculus* with a mean flowering day of 141 recorded from the iNaturalist dataset, and the predicted flowering day for *Sambucus* would be DOY 169, and the iNaturalist dataset shows a mean DOY of 166. Herbarium data spanning a period of 145 years predicts the mean flowering DOY for iNaturalist data in 2020, which suggests that the herbarium data and the iNaturalist data are recording comparable observations. However, many of the citizen science observations (which represent first flowering dates) are 3 weeks earlier, and a correction factor would be required to make the first flowering dates and peak flowering dates phenophases comparable.

### Geographical coverage

**Fig. 2:**
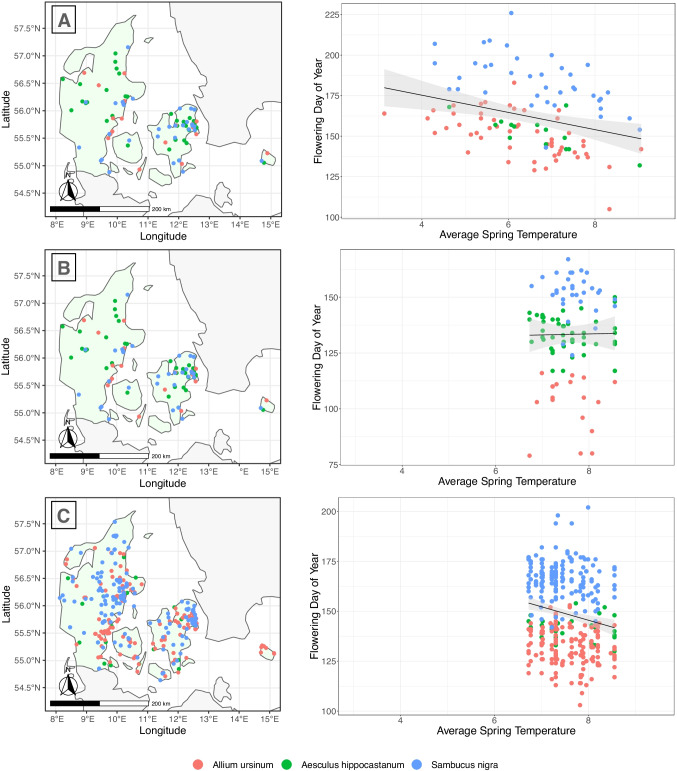
Distribution maps (left) and plots (right) of flowering day of year and average spring temperature (average temperatures for the months of March, April, and May) for *Allium ursinum*, *Aesculus hippocastanum*, and *Sambucus nigra* in Denmark (shown in light green). **A** the herbarium dataset (*n* = 110); **B** citizen science dataset (*n* = 104); **C** iNaturalist dataset (*n* = 403). Linear regression lines are shown in black and gray banding depicts 95% confidence interval (herbarium dataset [*R*^2^ = 0.09, *p* = 0.0014], citizen science dataset [*R*^2^ = 0.0001, *p* = 0.90], and iNaturalist dataset [*R*^2^ = 0.036, *p* = 0.00011]). Details of simple linear regression analyses for each of the three species in each dataset are presented in Supplemental information, S2

Each of the three phenology datasets has certain biases in distribution compared with the actual distribution of the three species in Denmark. The coverage of observations from the herbarium dataset is sparse or missing from the central and middle portions of the mainland (Jutland), where the species are common. *Aesculus* distribution is particularly poor in the herbarium dataset, and observations are clustered around the capital region of Copenhagen on the island of Zealand and virtually absent from the mainland (Fig. 2). The geographic coverage for the citizen science dataset is also poor, particularly around the middle of the mainland; however, contrary to the herbarium dataset, *Aesculus* is particularly well covered in the middle mainland (though absent from north and south mainland). *Allium* observations are lacking from the southwest of the country, and *Sambucus* observations are sparse from the northwest region of the country. The distribution of iNaturalist observations best represents the known distributions for these plants; however, observation density is highest around Denmark’s larger cities and towns (i.e., Copenhagen, Aarhus, Odense, and Kolding), and there are still large gaps in the coverage in the western part of the mainland.

Observation intensities and kernel density maps computed for each dataset in *spatstat* (Table 2 and Supplemental information, S2) illustrate the differences between species observations in each dataset and show that the iNaturalist dataset had the highest number of observations per unit area. Although all three datasets differ slightly in terms of their spatial biases, all datasets show biases of greater collecting around Denmark’s larger cities and towns. This is especially the case for the iNaturalist dataset (with four times the number of observations); however, this dataset shows a much more even distribution of observations, particularly around Jutland compared to the other datasets. Combining the herbarium dataset with the iNaturalist data improves the observation intensity.

**Table 2:** Comparison of the number of observations per unit area computed in *spatstat* for each of the three datasets, and the combined herbarium and iNaturalist data. The total number of observations is shown in parentheses. Intensity is a measure of the average density of points per unit area; the study area of Denmark was divided into 32 units. Two-way kernel density maps are presented in Supplemental information. Bold text represents the total values for each dataset

Dataset				
Species	Herbarium	Citizen science	iNaturalist	Combined
*Allium ursinum*	1.78 (57)	0.59 (19)	5.72 (183)	7.50 (240)
*Aesculus hippocastanum*	0.44 (14)	1.63 (52)	1.03 (33)	1.46 (47)
*Sambucus nigra*	1.22 (39)	1.03 (33)	5.84 (187)	7.06 (226)
Total intensity	**3.44 (110)**	**3.25 (104)**	**12.59 (403)**	**16.03 (513)**

### Spatial and temporal response of flowering

Annual mean temperature and average spring temperature (March, April, and May combined) in Denmark have increased significantly by a rate of 0.01 °C per year during the period 1872–2019 (Cappelen et al., 2020; see Supplemental information Figure S1). During this time period, the average spring temperature across Denmark (March, April, and May combined) ranged from 3.1 to 9.1 °C. For the year 2020, the average spring temperatures ranged from 6.7 to 8.6 °C across the country (Fig. 2); that is, the geographical variation in temperature in 2020 represented about one-third of the total variation in temperature found in the herbarium data set, which additionally includes inter-annual variation. Denmark spans a latitudinal range from 54 to 58° N and a longitudinal range from 8 to 15° E. A strong negative correlation was found between average spring temperature in 2020 and latitude (*r* =  − 0.76, *p* < 0.001), and a positive correlation was found between average spring temperature and longitude (*r* = 0.48, *p* < 0.001); that is temperatures are lower further north and east (see Supplemental information S5 for details).

*Herbarium dataset:* All three species were found to flower earlier with higher temperatures, but flowering did not become earlier over time, based on simple linear regressions of temperature or year on flowering DOY (data presented in Supplemental information, S3). Our best fit linear mixed model for the temperature data was a model with a flowering DOY as the response variable, species as a random effect, and average spring temperature and year as fixed effects, and the best-fitting model for the geographic variables included only longitude as a fixed effect (see Supplementary information, S4 for model comparison). The VIF values were all under 2 units, which indicates low multicollinearity between the predictor variables (climate, collection year, and geographic coordinates) is low (Zurr et al. 2010; see Supplementary information S4). Our linear mixed models show that average spring temperature is a strong predictor for flowering DOY (Table 3) and that for every increase of 1 °C in average spring temperature, flowering DOY advances by 7.4 ± 0.98 days (*p* < 0.001), although low marginal *R*^2^ values indicate that other factors than spring temperature and year explain variation in the flowering DOY.

**Table 3:** Linear mixed model results for the best fitting models for temperature models based on each of the three datasets: herbarium, citizen science, and iNaturalist, and the combined herbarium and iNaturalist data, with the flowering day of year (DOY) as the response variable, species as a random effect, and the following predictor variables as fixed effects in separate models: average spring temperatures (combined averages for March, April, and May), and year in the case of the herbarium dataset. SE = standard error. Details on the fit of the models and model selection are presented in Supplementary information (S4)

Dataset	Predictor variable	Regression coefficient	SE	*T*-value	*P*-value	Marginal *R*^2^	Conditional *R*^2^
Herbarium	Average spring (March–April–May)	− 7.40	0.98	− 7.58	< 0.001	0.13	0.76
	Year	0.05	0.03	1.46	0.15		
Citizen science	Average spring (March, April, and May)	− 2.23	1.75	− 1.28	0.21	0.001	0.88
iNaturalist	Average spring (March, April, and May)	− 2.69	0.87	− 3.10	0.002	0.01	0.78
Combined herbarium and iNaturalist	Average spring (March, April, and May)	− 5.21	0.60	− 8.70	< 0.001	0.04	0.80

*Citizen science dataset:* Simple linear regressions found no significant relationships between temperature and flowering time for all three species. The temperature model included flowering DOY as the response variable, species as a random effect, and average spring temperature as a fixed effect. The best-fitting model for the geographic variables was a model with only latitude as a fixed effect (see Supplementary information, S4). No significant relationship was found with average spring temperatures and flowering DOY in this dataset; that is, plants did not flower significantly earlier in areas where higher temperatures were recorded—or the variation in temperature captured from a single season for this dataset was not high enough to confidently predict flowering DOY. The much lower marginal *R*^2^ values seen for this dataset compared to the herbarium models may indicate that this dataset not only lacked in temporal variation but also lacked in the number of observations to capture sufficient spatial variation to explain flowering DOY. However, based on the very high conditional *R*^2^ values and low marginal *R*^2^ values, latitude only weakly explains DOY in this dataset (Table 4).

**Table 4:** Linear mixed model results for the best fitting models for geographic models for each of the three datasets, with flowering day of year (DOY) as the response variable, species as random effect, geographical coordinates (longitude and latitude) as fixed effects. SE = standard error. Details on the fit of the models and model selection are presented in Supplementary information (S4)

Dataset	Predictor variable	Regression coefficient	SE	*t*-value	*P*-value	Marginal R^2^	Conditional R^2^
Herbarium	Longitude	− 1.50	0.96	− 1.60	0.12	0.01	0.52
Citizen science	Latitude	3.00	1.20	1.50	0.128	0.004	0.83
iNaturalist	Longitude	− 0.89	0.35	− 2.50	0.0125	0.005	0.71
Combined herbarium and iNaturalist	Longitude	− 1.10	0.35	− 3.00	0.00255	0.004	0.76

*iNaturalist dataset:* Simple linear regressions found that average spring temperature was a strong predictor for flowering time in *Allium*, and average April temperature and average May temperature were found to significantly explain flowering based on *p*-values in *Aesculus* and *Sambucus* respectively (Supplemental information S3). The temperature model for the iNaturalist data included flowering DOY as the response variable, species as a random effect, and average spring temperature as a fixed effect, and for the geographic model, only longitude was included as a fixed effect. In contrast to the citizen science dataset also collected over a single year, average spring temperature was found to be a significant predictor of flowering DOY based on *p*-values; however, based on low *R*^2^ values, the temperature is considered only a weak predictor (Table 3). For every 1 °C increase in average spring temperature, flowering DOY advances by 2.7 ± 0.9 days (*p* = 0.002). Marginal *R*^2^ values are, however, much lower than seen in the herbarium dataset, indicating that additional environmental variables are missing from our models to explain variation in flowering phenology. In addition, the best model run with geographic coordinates as fixed effects shows that longitude also explains flowering DOY in this dataset. Flowering day of year decreases significantly by 0.9 days per increase in longitudinal degree across Denmark (Table 4). The very low *R*^2^ values likely indicate that longitudinal change in Denmark is probably obscured by changes in other environmental conditions, such as precipitation, underlying geology, or even genotypic differences for example (Iwanycki Ahlstrand et al. 2018).

*Combined herbarium and iNaturalist datasets:* The best fit linear mixed model for temperature and the combined herbarium and iNaturalist datasets included species and dataset as random effects and average spring temperature as a fixed effect, and for geographic coordinates, a model with only longitude as a fixed effect. The combined data includes the largest number of observations of data with good temporal and spatial coverage. This increased the slope of the regression line and improved the confidence in the relationship between temperature and flowering time based on *p*-values; however, although marginal *R*^2^ values were higher than the iNaturalist data alone, they were much lower than the herbarium data alone (Table 3, Supplemental information S6). For every 1 °C increase in average spring temperature, flowering DOY advances by 5.22 ± 0.6 days (*p* < 0.001). The model run with geographic coordinates as fixed effects show that longitude explains flowering DOY in this combined dataset, where the flowering day of year decreases by 1.06 ± 0.35 days (*p* < 0.05) per increase in longitudinal degree, or from west to east across Denmark (Table 4).

## Discussion

### Inter-annual variation better predicts phenological shifts than geographical coverage

In this study, we compared three different datasets that measure different ecological phenomena: herbarium data that captures long-term or temporal variation and two contrasting sets of citizen science data that capture spatial variation in flowering time. Geographic coverage over Denmark, as measured by latitude and/or longitude, was found to be important in detecting shifts in phenology for the citizen science dataset and the iNaturalist dataset but was not a surrogate for phenology data gathered over many years. A small herbarium dataset (*n* = 110), capturing close to 145 years of inter-annual variation, was found to detect the greatest phenological shifts based on modeling the relationship between flowering day of year and spring temperature. The trends we obtained from herbarium data are comparable to findings of other phenology studies based on long-term field-collected data in the UK and elsewhere in Europe for the same species, with flowering advancing 6–7 days per increase in degrees Celsius (Sparks et al. 2000; Menzel 2003). The importance of long time series for studying the phenological response to climate has long been recognized, and numerous studies have now demonstrated the value of herbarium specimens in recreating long-term phenology data and advancing the study of global change (Primack et al. 2004; Calinger et al. 2013; Davis et al. 2015; Lang et al. 2019). Citizen science data of equal sample size to our herbarium data from a directed phenology monitoring program detected no trends between flowering DOY and climate. One year of sampling data, with a relatively low number of observations from across the country is not sufficient to capture the amount of year-to-year climatic variation found in our study region based on historical climatic fluctuations. This may be particularly true in a small region with relatively little topographic variation, such as Denmark. The range of topographic variation was further limited in the citizen science data set by a biased collection of observations around Copenhagen and other large cities. However, if observations from a single year are numerous enough, and have a more complete geographic coverage, as was seen in our iNaturalist dataset, significant shifts in flowering with respect to climate and geographic location are detectable, though not to the same extent as with the herbarium data collected over long time spans (2 days per degree Celsius for iNaturalist data versus 7 days per degree Celsius for herbarium data). The iNaturalist dataset, in particular, demonstrates that spatial variation may be confounded with other important variables that influence flowering time and deserve further investigation. One of the most important findings of this study is that phenology data from iNaturalist and from herbarium specimens are comparable in terms of documenting peak flowering times and for determining the relationship between spring temperature and flowering times, and if combined, can improve our understanding of both spatially and temporally important variables in explaining the impact of climate change on plant phenology.

### Geographic biases

While the herbarium dataset was effective at determining temperature effects on flowering times, the herbarium specimens had major geographic biases in the collection as found in other studies (Delisle et al., 2003; Daru et al. 2018). Geographical biases of herbarium specimens may be even greater for the types of common species used in our study, as these species are likely to be overlooked by botanists traveling to more distant collecting sites and looking for rare species. The statistical power of using herbarium specimens to study climate change may further be weakened by the inability to assign precise locations to many specimens (Bloom et al. 2017; Kosanic et al. 2018). Also, the ability to measure species’ phenological response to local weather is limited by the small number of weather stations available to estimate temperature on a finer geographical scale (i.e., only 5 stations in Denmark’s recorded historical data, compared to 45 weather stations in 2020, Supplemental Information, Figure S1.2).

Geographic biases associated with the herbarium and citizen science datasets may further influence the detection of other important variables in explaining flowering times. For example, latitude and longitude have been shown to influence flowering phenology in Europe and elsewhere in northern latitudes (Estella et al. 2009; Wang et al. 2015; Gao et al. 2020). We show that by combining the herbarium dataset with the iNaturalist dataset and improving both geographic coverage and the number of observations, thereby capturing greater environmental variation in Denmark, longitude becomes a stronger predictor and increases in their value in explaining flowering times. We attribute this to a change in environmental conditions associated with geography in Denmark, such as changes in precipitation, in geology, and potentially genotypic or historical differences resulting from the disjunct terrestrial land and/or islands that are included in Denmark (Iwanycki Ahlstrand et al. 2018).

### Peak versus first flowering day of year

We found a strong link between flowering DOY and spring temperature in both the herbarium data and the iNaturalist data: a regression equation calculated based on herbarium data was able to predict a mean flowering DOY based on average spring temperatures recorded for the year 2020 which matched our iNaturalist data. This finding supports our assumption that phenology data derived from the herbarium and iNaturalist data represent the same phenological stage of peak flowering. This result also further supports the idea that incidental iNaturalist observations may be comparable to museum vouchers in phenological studies and even that herbarium specimen data and iNaturalist data could be combined for certain statistical purposes (Heberling and Isaac 2018; iNaturalist, 2020). iNaturalist data is comparable to data from historical collections of photographs which have also been used in past climate change studies (Miller-Rushing et al. 2006; Panchen et al. 2012).

The citizen science data used in our study differs from the herbarium specimen data and the iNaturalist data by specifically recording the first flowering day of year. The first flowering day of year has been used as the preferred phenophase metric by many as the first flowering day of year has been found to be correlated with peak flowering, and less effort is required to collect such data (Fitter and Fitter 2002; Molau et al. 2005; Fox and Jönsson 2019). However, first event dates may be less effective in some climate change studies due to their sensitivity to changing population size, plant size, and sampling intensity (Miller-Rushing et al. 2009; Smith and Ramsay 2020). Our inability to detect a relationship between the first flowering day of year and temperature could be due to an inability to account for these factors, along with a lower range of temperatures and another environmental variability in comparison with the other two datasets. Statistical methods have been proposed for combining the first observation of the season with peak observations in phenology studies, however, the practical value of such approaches has not been settled (Pearse et al. 2017; Miller et al. 2021).

### Future citizen science phenology initiatives

The historical nature of herbarium specimens makes them invaluable as a data source for studying the effects of climate change over the past century. In addition, new citizen science initiatives, both through directed program or incidental biodiversity observations, have huge potential to expand the geographical intensity and sample size of climate change studies and improve knowledge of species-specific responses, even though these studies have a short time span. We therefore encourage the continued development of new initiatives, especially in Nordic countries and northerly latitudes where long-term phenology studies are sparse. These new citizen science programs may provide the best field approach to investigate key environmental variables that affect phenology, including photoperiod, temperature, and precipitation (Wolkovich, Cook, and Davies 2013; Tang et al. 2016). Programs involving hundreds or even thousands of observers have the potential to capture highly detailed, accurate, and widely distributed dates for phenological events of individual species, in a manner not captured by other approaches, such as herbarium studies, botanical gardens studies, and remote sensing. Our result confirms that citizen scientists can provide useful phenology data when phenology events are selected that are distinct and easily recognized, in the case of citizen science programs, or that can be confirmed by expert evaluation, such as the iNaturalist data (Fuccillo Battle et al. 2014; Bison et al. 2019).

Based on our findings for a single year of observations in Denmark, we recommend that even if broad geographic coverage is achieved, a minimum of several years of citizen science data needs to be accumulated to ensure that inter-annual fluctuations of warm years and cool years are reflected in the data. Where possible, geographic coverage should at minimum be targeted to reflect both known species distributions for the targeted organisms, as well as the highest resolution available climate data. In situations where there is a much greater degree of climatic variation within a region or country, such as Italy or Acadia National Park in the USA, fewer years might be adequate to detect relationships between temperature and phenology (MacKenzie et al. 2017). Similarly, larger number of observations might allow patterns to be detected in fewer years.

For directed citizen science data obtained from programs such as iNaturalist to be combined with herbarium (and other) phenology data, peak flowering day of year should be emphasized as the key phenological phase to be observed, or alternatively, multiple phenophases from the same plants could be monitored to compare with herbaria records (i.e., first flowering, peak flowering, and fruiting). However, the number of phenophases to observe being included in instructions to citizen scientists needs to be tempered with data quality. In a recent study comparing trained citizen scientists to untrained volunteers, it was found that biases were introduced by repeated phenological observations and that having citizen scientists make one observation may produce data as good if not better than individuals making repeated observations (Feldman et al. 2018). The particular advantage of iNaturalist data is that even though people are taking pictures throughout a plant’s phenological stages, most photos are taken when plants are in full flower, which is precisely comparable to when herbarium specimens are likely to be collected. Previous studies have shown that herbarium specimens, photograph collections, and field observations of plants in full flower can be effectively combined in climate change studies (Primack et al. 2004; Panchen et al. 2012).

A great advantage of iNaturalist data is that the identity of the plant can be checked against the accompanying photograph (Heberling and Issac 2018; iNaturalist, 2021; Putman et al. 2021). Not only does this allow misidentified plants to be eliminated from the data set, but it allows cultivars to be eliminated as well as plants growing in garden settings. In contrast, with citizen science programs that send people out into nature to make observations, there is typically no way to check if a participant is looking at the correct species, and a surprisingly high percentage of observations might involve misidentified plants (MacKenzie et al. 2017).

## Conclusions

This study examined three distinct data sources for the flowering phenology of three common plants in Denmark. We found that data collected from herbarium specimens provided the strongest signal of phenological response to temperature. Data collected from people using iNaturalist also provided comparable information, and these observations could be combined with data gathered from herbarium specimens to capture both spatial and temporal patterns, as well as increase the number of observations for climate change analysis. In contrast, data on first flowering collected through a directed citizen science network was not able to detect any environmental effects on first flowering, likely due to smaller sample size, a more limited geographical distribution, and the confounding effects of sample biases.

In the coming years, the sample sizes of directed citizen science programs and iNaturalist observations will likely continue to expand and will incorporate the natural climatic variation from year to year. The organizers of these programs should encourage people to participate in these programs and urge people to make observations in parts of the country which are under-represented in the dataset and/or will maximize climatic variation. When these programs have additional years of data, and their observations are combined with data from herbarium specimens, scientists will have an increasingly powerful tool for investigating climate change in Denmark.

## Supplementary Information

Below is the link to the electronic supplementary material.

Supplementary file1 (PDF 587 KB)

## Data Availability

Datasets assembled for this study will be made publicly available as Supplemental information, and species/specimen occurrence data will be published to GBIF upon acceptance of the manuscript.
