# Correction to: A comparison of herbarium and citizen science phenology datasets for detecting response of flowering time to climate change in Denmark

**DOI:** 10.1007/s00484-022-02272-8

**Published:** 2022-03-14

**Authors:** Natalie Iwanycki Ahlstrand, Richard B. Primack, Anders P. Tøttrup

**Affiliations:** 1 Natural History Museum of Denmark, University of Copenhagen, Copenhagen, Denmark; 2 Biology Department, Boston University, Boston, MA USA

**Correction to: Space Science Reviews**

**https://doi.org/10.1007/s00484-022-02238-w**

Due to figure processing errors introduced during typesetting via the publisher, the article was originally published with Figure 1 missing and Figure 2 appearing twice (in place of Fig. 1 and in its own respective place).

This correction stands to support the update to the original article. The original article has been corrected.

