# Supplementary material for: A comparison of herbarium and citizen science phenology datasets for detecting response of flowering time to climate change in Denmark
Source: Int J Biometeorol. 2022 Mar 2;66(5):849–62. doi: 10.1007/s00484-022-02238-w (PMC9042978; doi:10.1007/s00484-022-02238-w)
Supplement: Supplementary file 1 — Supplementary file1 (PDF 587 KB) [file 484_2022_2238_MOESM1_ESM.pdf]

Supplemental Information

A comparison of herbarium and citizen science phenology datasets for detecting response of flowering time to climate change in Denmark

Natalie Iwanycki Ahlstrand, Richard B. Primack, Anders P. Tøttrup

Contents:

S1. Climatic variables over time in Denmark
S2. Kernel Density Maps
S3. Simple linear regression analyses
S4. Linear Mixed Model Selection
S5. Variance Inflation Factor and the correlation of environmental variables
S6. Combined herbarium and iNaturalist datasets

S1. Climatic variables over time in Denmark

The annual mean temperature in Denmark has increased significantly by 0.01 º C per year during the period 1872–2019 (Cappelin et al., 2020; see Supplemental Information **Figure S1**).

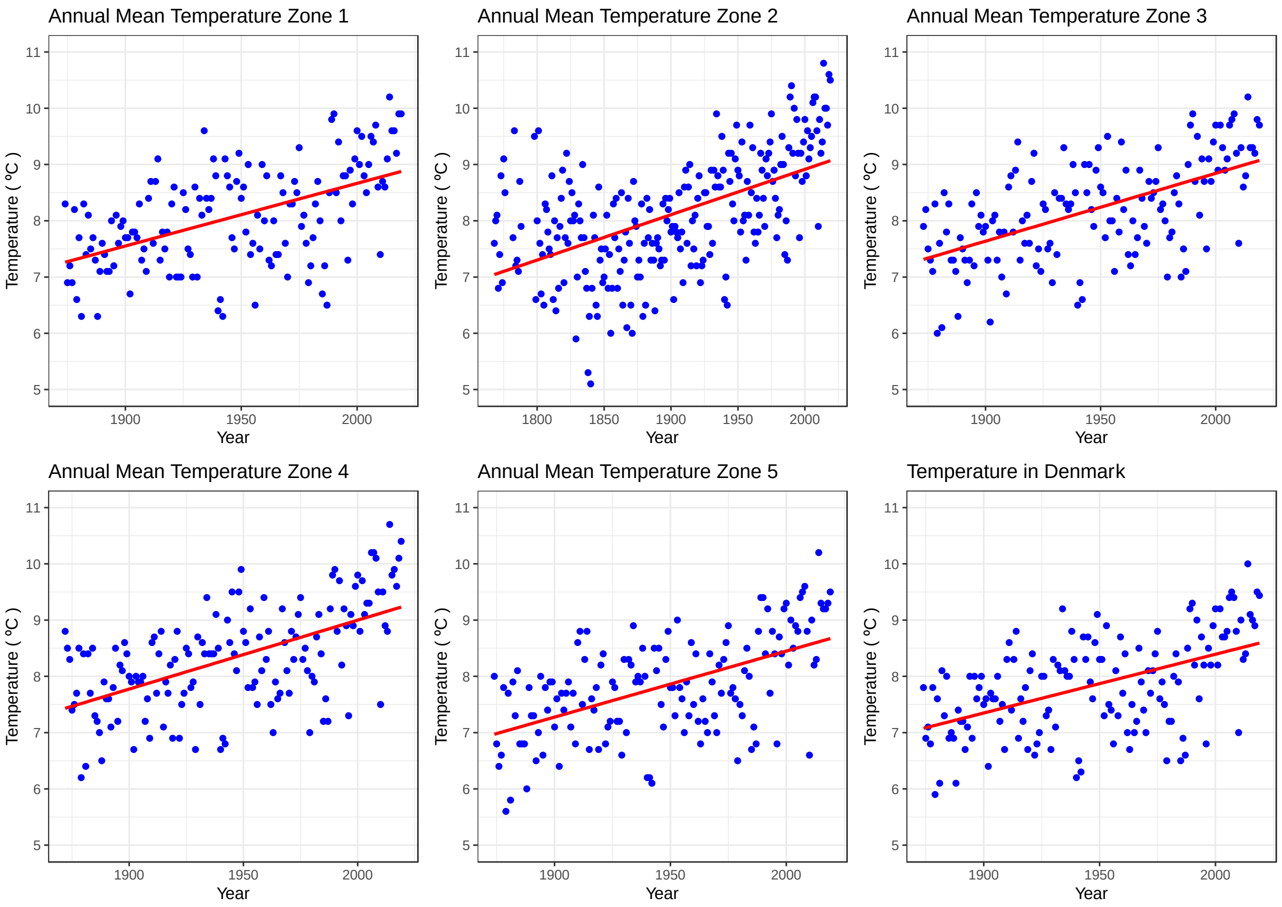

Figure S1.1. Annual mean temperature data from 1872-2019 for the five weather Danish Meteorological Institute stations recording historical climate data in Denmark (Zone 1 to 5), and the average annual mean temperature from all five stations across Denmark. Linear regression analyses for the six plots show increases in annual temperature at a rate of 0.01 º C per year: Zone 1 increase of 0.011 º C per year (R^2= 0.2757 , p = 1.02^{e-11}); Zone 2 increase of 0.08 º C per year (R^2 = 0.2882, p = < 2^{e-16}); Zone 3 increase of 0.012 º C per year (R^2 = 0.3298, p = 2.86^{e-14}); Zone 4 increase of 0.0122 º C per year (R^2 = 0.3203, p = 6.60^{e-14}); Zone 5 increase of 0.012 º C per year (R^2 = 0.3019, p = 6.85^{e-13}); Average for all weather stations increase of 0.01 º C per year (R^2 = 0.2632, p = 3.58^{e-11}).

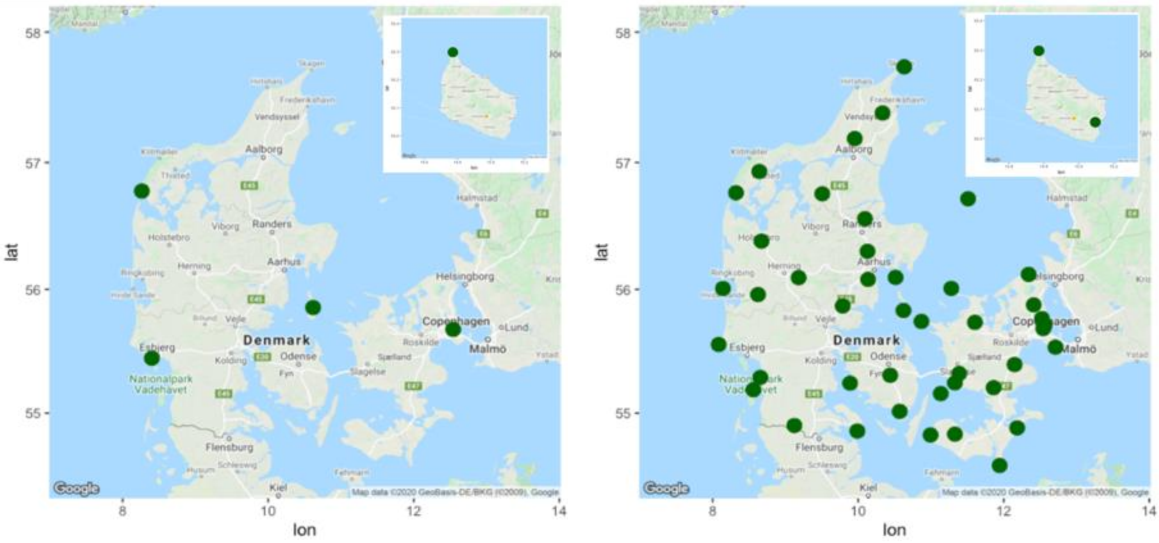

Figure S1.2. Locations of DMI weather stations in Denmark. A) historical stations; B) weather stations providing data in 2020.

S2. Kernel Density Maps

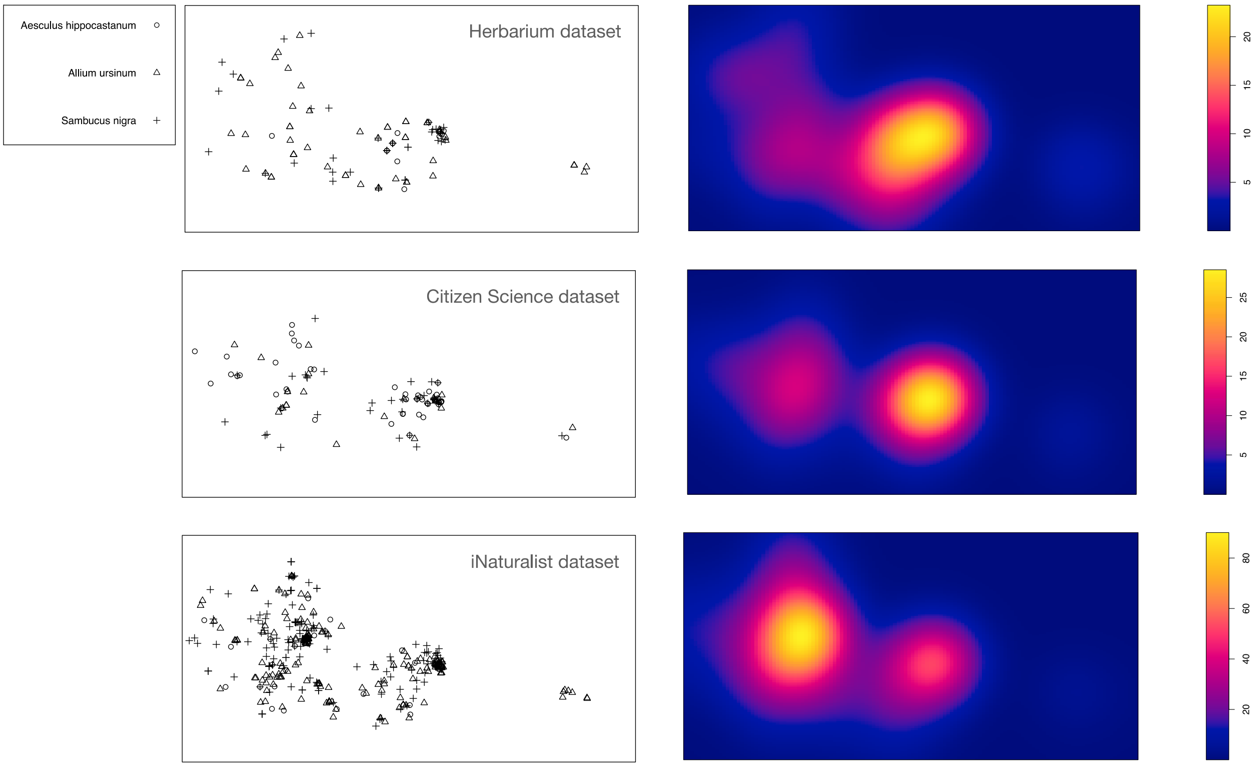

Figure S2.1 Two-dimensional kernel density maps computed in the R package *spatstat* for each dataset demonstrating spatial biases and differences in sampling intensities. From top to bottom, herbarium dataset, citizen science dataset, iNaturalist dataset.

S3. Simple linear regression analyses

Simple linear regressions were run individually for each species and each dataset, using “flowering day of year” as a response variable, and climatic variables as explanatory variables (average temperatures in the months of March, April and May, and “average spring temperature”, combining the monthly averages for March, April, and May into a spring average).

Herbarium dataset

Year was not found to be a good predictor of flowering time. During time period from 1846–1991, the flowering DOY derived from our herbarium dataset decreased for *Allium*, though not significantly, by 0.05 days per year, but increased, also not significantly, for *Aesculus* and *Sambucus*. However, for all three species, flowering DOY was found to decrease significantly with higher spring temperatures (**Figure S2**). Average spring temperature (combined averages for March, April and May) was found to be the best predictor of flowering day of year for each of three focal species, and R^2 values were higher for combined average spring than for any individual months; however different individual months were found to be better predictors of flowering day of year (Supplemental Material **Table S2.1**). For example, April was the best predictor for *Allium ursinum* and *Aesculus hippocastanum*, and but both the months of April and May were equally good predictors for *Sambucus nigra*.

Citizen Science dataset

No significant relationships were found between flowering time observations in our citizen science dataset and climatic variables (Table S2.2).

iNaturalist dataset

Simple linear regression found no significant relationships for *Aesculus* flowering day of year and climatic variables. However, for *Allium*, average spring temperature was found to be significant (p =0.00384), as well as average the individual monthly temperatures for March, April temperature, and May. For *Sambucus*, only average May temperature was found to have a slight significant relationship (Table S2.3).

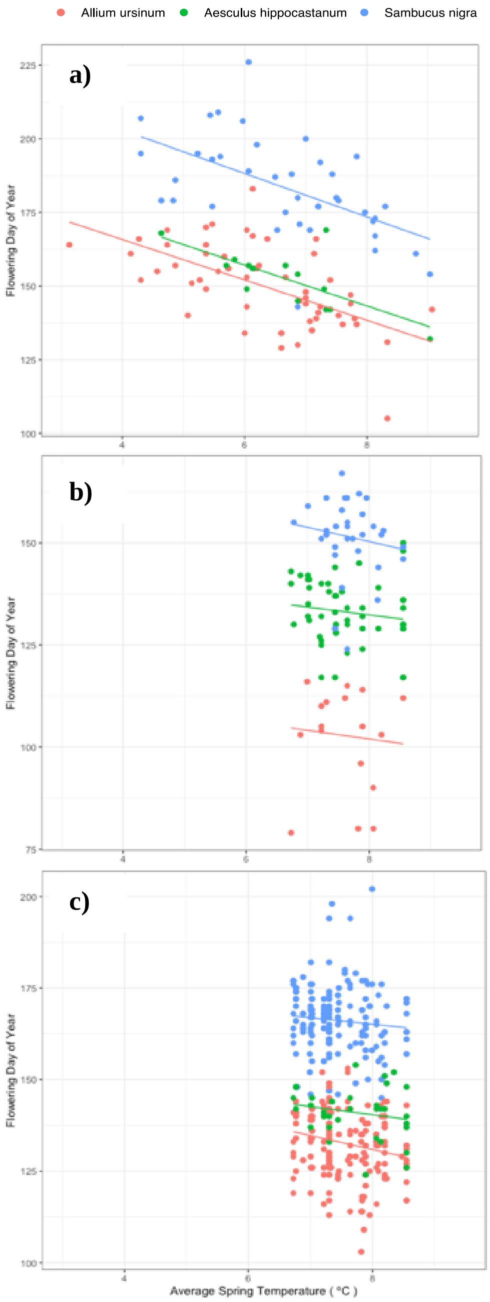

Figure S3.1. Simple linear regression analyses for *Allium ursinum*, *Aesculus hippocastanum*, and *Sambucus nigra*, in the three separate datasets **a)** herbarium dataset; **b)** citizen science dataset; **c)** iNaturalist dataset. Regression lines show the relationship between flowering day of year and average spring temperature for each species.

Table S3.1 Herbarium Data. Linear Regression Analyses of flowering day of year and environmental and temporal variables.

**Predictor variable**	**Co-efficient**	**S.E.**	***t*-value**	***P*-value**	R^2	**Adj** R^2	***F*-stat**
Allium ursinum **(*n*=57)**							
Average Spring	-6.85	1.26	-5.42	**1.35e-06**	0.35	0.34	29.41
Average March	-3.34	0.97	-3.44	**0.001102**	0.18	0.16	11.86
Average April	-5.17	1.16	-4.47	**4e-05**	0.267	0.252	19.95
Average May	-3.63	1.07	-3.41	**0.00124**	0.17	0.16	11.6
Year	-0.05	0.05	-1.04	0.3038	0.02	0.00	1.08
Aesculus hippocastanum **(*n*=13)**							
Average Spring	-6.93	1.96	-3.53	**0.00416**	0.51	0.47	12.45
Average March	-2.71	1.65	-1.64	0.128	0.18	0.11	2.68
Average April	-5.39	1.61	-3.35	**0.00577**	0.48	0.44	11.23
Average May	-5.46	2.17	-2.52	0.0272	0.35	0.29	6.33
Year	0.03	0.08	0.31	0.762	0.00	-0.07	0.10
Sambucus nigra **(*n*=35)**							
Average Spring	-7.37	1.78	-4.13	**0.000199**	0.32	0.30	17.06
Average March	-3.97	1.11	-3.58	**0.000975**	0.26	0.24	12.83
Average April	-3.23	1.78	-1.82	0.0763	0.08	0.06	3.33
Average May	-7.20	1.93	-3.74	**0.000628**	0.27	0.25	13.96
Year	0.03	0.07	0.37	0.7175	0.00	-0.02	0.13

Table S3.2. Citizen Science Data. Linear Regression Analyses of flowering day of year and environmental variables.

**Predictor variable**	**Co-efficient**	**S.E.**	***t*-value**	***P*-value**	R^2	**Adj** R^2	***F*-stat**
***Allium ursinum* (*n*=19)**							
Average Spring	-2.12	5.94	-0.36	0.7259	0.01	-0.05	0.13
Average March	-2.34	6.85	-0.34	0.73716	0.01	-0.05	0.12
Average April	-2.36	5.73	-0.41	0.686	0.00	-0.05	0.17
Average May	-1.46	5.00	-0.29	0.774	0.00	-0.05	0.09
***Aesculus hippocastanum* (*n*=52)**							
Average Spring	-1.91	1.85	-1.03	0.31	0.02	0.00	1.06
Average March	- 2.21	2.31	-0.96	0.343	0.02	0.00	0.92
Average April	-1.61	1.77	-0.91	0.37	0.02	0.00	0.82
Average May	-1.83	1.56	-1.17	0.249	0.03	0.00	1.36
***Sambucus nigra* (*n*=33)**							
Average Spring	-3.47	4.02	-0.86	0.395	0.02	-0.00	0.75
Average March	-2.77	4.31	-0.64	0.526	0.01	-0.01	0.41
Average April	-2.74	3.83	-1.00	0.317	0.00	0.00	1.01
Average May	-4.02	3.60	-1.12	0.272	0.04	0.01	1.25

Table S3.3. iNaturalist Data. Linear Regression Analyses of flowering day of year and environmental variables.

Predictor variable	Co-efficient	S.E.	*t*-value	*P*-value	R^2	Adj R^2	*F*-stat
***Allium ursinum* (*n*=183)**							
Average Spring	-3.82	1.30	-2.93	**0.00384**	0.05	0.04	8.58
Average March	-3.46	1.54	-2.45	**0.0259**	0.03	0.02	5.05
Average April	-4.06	1.27	-3.20	**0.00164**	0.05	0.05	10.22
Average May	-3.37	1.10	-3.05	**0.0026**	0.05	0.04	9.33
***Aesculus hippocastanum* (*n*=33)**							
Average Spring	-2.24	1.96	-1.14	0.262	0.04	0.01	1.30
Average March	-1.37	2.29	-0.60	0.554	0.01	-0.02	0.36
Average April	-2.78	1.94	-1.43	**0.000226**	0.06	0.03	2.06
Average May	-2.09	1.66	-1.26	0.217	0.05	0.02	1.59
***Sambucus nigra* (*n*=187)**							
Average Spring	-1.77	1.35	-1.30	0. 20	0.01	0.00	1.69
Average March	-0.68	1.55	-0.44	0.66	0.00	0.00	0.19
Average April	-1.35	1.34	-1.00	0.317	0.00	0.00	1.01
Average May	-2.38	1.13	-2.01	**0.0369**	0.02	0.02	4.42

S.4 Linear Mixed Model Selection

Table S.4.1 Selection of the best fitting linear mixed model for the herbarium dataset was based on the lowest Akaike Information Criterion (AIC) value. The first two models include flowering day of year (DOY) as response variable, and temperature and year as fixed effects. Models 3, 4 and 5 include flowering DOY as response variable, and geographic variables (longitude and latitude) as fixed effects. Best fit models shown in *italics*.

**Model No.**	**Model**	**AIC**	**BIC**	**Log-likelihood**	**AICc**	***R^2 Marginal/ Conditional***
**1**	DOY ~ averageSpring + (1|Species)	874.4	885.2	-433.2	874.78	0.16/0.7
2	*DOY ~ averageSpring+year + (1|Species)*	*874.24*	*887.74*	*-432.12*	*874.82*	*0.16/0.7*
3	*DOY ~ longitude + (1|Species)*	*916.59*	*927.39*	*-454.29*	*916.97*	*0.01/0.52*
**4**	DOY ~ latitude + (1|Species)	918.92	929.72	-455.46	919.30	0.01/0.52
**5**	DOY ~ latitude+longitude + (1|Species)	917.71	931.21	-453.86	918.29	0.02/0.53

Table S.4.2. Selection of the best fitting linear mixed model for the citizen science dataset was based on the lowest Akaike Information Criterion (AIC) value. The first model includes flowering day of year (DOY) as response variable, and temperature as a fixed effect. Models 2, 3 and 4 include flowering DOY as response variable, and geographic variables (longitude and latitude) as fixed effects. Best fit model shown in *italics*.

**Model No.**	**Model**	**AIC**	**BIC**	**Log-likelihood**	**AICc**	***R^2 Marginal/ Conditional***
1	*DOY ~ averageSpring + (1|Species)*	*776.23*	*786.81*	*-384.12*	*776.64*	*0.002/0.83*
**2**	DOY ~ longitude + (1|Species)	777	787.57	-384.5	778.76	0.001/0.83
3	*DOY ~ latitude + (1|Species)*	*775.52*	*786.1*	*-383.76*	*777.90*	*0.004/0.83*
**4**	DOY ~ latitude+longitude + (1|Species)	777.45	790.67	-383.72	780.15	0.004/0.83

Table S.4.3. Selection of the best fitting linear mixed model for the iNaturalist dataset was based on the lowest Akaike Information Criterion (AIC) value. The first model includes flowering day of year (DOY) as response variable, and temperature as a fixed effect. Models 2, 3 and 4 include flowering DOY as response variable, and geographic variables (longitude and latitude) as fixed effects. Best fit model shown in *italics*.

**Model No.**	**Model**	**AIC**	**BIC**	**Log-likelihood**	**AICc**	***R^2 Marginal/ Conditional***
1	*DOY ~ averageSpring + (1|Species)*	*2942.5*	*2958.49*	*-1467.2*	*2942.6*	*0.01/0.71*
2	*DOY ~ longitude + (1|Species)*	*2945.8*	*2961.8*	*-1468.9*	*2945.87*	*0.004/0.71*
**3**	DOY ~ latitude + (1|Species)	2946.8	2962.8	-1469.4	2946.89	0.004/0.71
**4**	DOY ~ latitude+longitude + (1|Species)	2945.8	2965.8	-1467.9	2945.91	0.01/0.71

Table S.4.4. Selection of the best fitting linear mixed model for the combined herbarium and iNaturalist datasets was based on the model with the lowest Akaike Information Criterion (AIC) value. The first and second models include flowering day of year as response variable, and temperature and year as fixed effects. Models 3, 4 and 5 and 6 include flowering DOY as response variable, and geographic variables (longitude and latitude) and year as fixed effects.

**Model No.**	**Model**	**AIC**	**BIC**	**Log-likelihood**	**AICc**	***R^2 Marginal/ Conditional***
1	*DOY ~ averageSpring + (1|Species) + (1|Dataset)*	*3831.5*	*3852.7*	*-1910.7*	*3831.59*	*0.05/0.75*
**2**	DOY ~ averageSpring+year + (1|Species) + (1|Dataset)	3832.4	3857.8	-1910.2	3832.54	0.04/0.76
3	*DOY ~ longitude + (1|Species) + (1|Dataset)*	*3892.7*	*3913.9*	*-1941.3*	*3892.8*	*0.04/0.76*
**4**	DOY ~ latitude + (1|Species) + (1|Dataset)	3899.6	3920.8	-1944.8	3899.70	0.001/0.76
**5**	DOY ~ longitude+latitude + (1|Species) + (1|Dataset)	3894.6	3920	-1941.3	3894.72	0.004/0.76
**6**	DOY ~ year+longitude+latitude + (1|Species) + (1|Dataset)	3896.3	3926	-1941.2	3896.54	0.005/0.75

S5. Calculating multicollinearity and the correlation of environmental variables

Variance Inflation Factors

Variance Inflation Factors (VIF) were calculated for each model using the car package in R to test for multicollinearity in the variables included in our models for each of the three datasets (ref). Additionally, Pearson correlations were calculated for the variables in each dataset. In all cases, VIF calculations did not exceed 5 for any of the variables used in our models (see Table XXX).

Table S5.1 Herbarium dataset: Variance Inflation Factors (VIF) calculated for variables used in our herbarium dataset models 1, 2, 3, 4 and 5 (details above, Table S4.1). VIF values cannot be calculated for models that do not include more than one explanatory variable/ fixed effect.

Variable	Model 1	Model 2	Model 3	Model 4	Model 5
Average Spring	–	1.07	–	–	–
Year	–	1.07	–	–	–
Longitude	–	–	–	–	1.15
Latitude	–	–	–	–	1.15

Table S5.2 Citizen Science dataset: Variance Inflation Factors (VIF) calculated for variables used in our herbarium dataset models 1, 2, 3, and 4 (details above, Table S4.2). VIF values cannot be calculated for models that do not include more than one explanatory variable/ fixed effect.

Variable	Model 1	Model 2	Model 3	Model 4
Longitude	–	–	–	1.19
Latitude	–	–	–	1.19

Table S5.3 iNaturalist dataset: Variance Inflation Factors (VIF) calculated for variables used in our herbarium dataset models 1, 2, 3, and 4 (details above, Table S4.3). VIF values cannot be calculated for models that do not include more than one explanatory variable/ fixed effect.

Variable	Model 1	Model 2	Model 3	Model 4
Average Spring	–	1.42	1.5	–
Longitude	–	1.42	–	1.47
Latitude	–	–	1.5	1.56

Table S.5.4. Combined herbarium and iNaturalist datasets: Variance Inflation Factors (VIF) calculated for variables used in our herbarium dataset models 1, 2, 3, 4, 5 and 6 (details above, Table S4.4). VIF values cannot be calculated for models that do not include more than one explanatory variable/ fixed effect.

Variable	Model 1	Model 2	Model 3	Model 4	Model 5	Model 6

Average Spring	–	1.04	–	–	–	–
Year	–	1.04	–	–	–	1.0
Longitude	–	–	–	–	1.18	1.18
Latitude	–	–	–	–	1.18	1.18

Pearson correlations analyses

For our herbarium dataset and the historical weather data, average spring temperature was positively correlated with longitude (not significant), and negatively correlated with latitude (not significant) (Table S5.5). Strong negative correlations were found between average temperatures in March, April, and May with latitude, and positive correlations were found with longitude for both our citizen science and iNaturalist datasets (Table S5.6 and S5.7). For the combined herbarium and iNaturalist dataset, a strong negative correlation was found between temperature and latitude, but a non-significant positive correlation with longitude (Table S5.8)

Table S5.5 Herbarium dataset: Pearson correlations for average spring temperatures (combined averages for the months of March, April, May) and environmental variables.

**Variable**	**Co-efficient**	***t***	***p*-value**
Year	0.24	2.58	0.01
Latitude	-0.71	-0.74	0.46
Longitude	0.11	1.19	0.24

Table S5.6 Citizen Science dataset: Pearson correlations for average spring temperatures (combined averages for the months of March, April, May) and environmental variables.

**Variable**	**Co-efficient**	***t***	***p*-value**
Latitude	-0.76	-23.73	<0.001
Longitude	0.48	11.02	<0.001

Table S5.7 iNaturalist dataset: Pearson correlations for average spring temperatures (combined averages for the months of March, April, May) and environmental variables.

**Variable**	**Co-efficient**	***t***	***p*-value**
Latitude	-0.57	-7.07	<0.001
Longitude	0.54	6.53	<0.001

Table S5.8 Combined Herbarium and iNaturalist dataset: Pearson correlations for average spring temperatures (combined averages for the months of March, April, May) and environmental variables.

**Variable**	**Co-efficient**	***t***	***p*-value**
Latitude	-0.29	-0.37	< 0.001
Longitude	0.16	3.84	0.25

S6. Combined herbarium and iNaturalist datasets

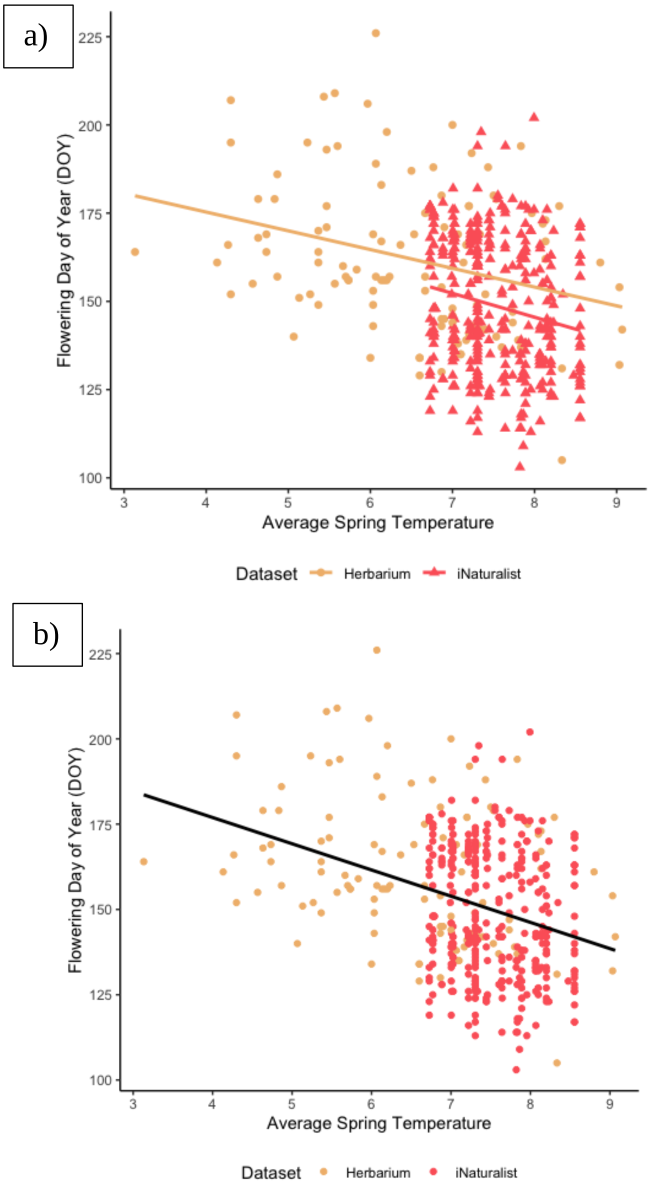

Figure S6.1 Plots of average spring temperature and flowering day of year from combined herbarium and iNaturalist datasets. A) Linear regression line shown for each dataset; b) linear regression line shown for combined dataset. Combined dataset: -7.68 ± 0.99, t = -7.71, R^2 = 0.1 $p < 0.001$; Herbarium data: -5.31 ± 1.67, t = -7.71, R^2 = 0.09 p = 0.0014; iNaturalist data: -6.45 ± 1.46, t = -4.43, R^2 = 0.04, $p < 0.001$). See Tables 3 and S4.1 for results of linear mixed models.
